# Structural Analysis of Phosphoserine Aminotransferase (Isoform 1) From *Arabidopsis thaliana*– the Enzyme Involved in the Phosphorylated Pathway of Serine Biosynthesis

**DOI:** 10.3389/fpls.2018.00876

**Published:** 2018-07-06

**Authors:** Bartosz Sekula, Milosz Ruszkowski, Zbigniew Dauter

**Affiliations:** Synchrotron Radiation Research Section, Macromolecular Crystallography Laboratory, National Cancer Institute, Argonne, IL, United States

**Keywords:** serine metabolism, PSAT, PLP, pyridoxal 5′-phosphate, transaminase, geminal diamine

## Abstract

Phosphoserine aminotransferase (PSAT) is a pyridoxal 5′-phosphate (PLP)-dependent enzyme that catalyzes the conversion of 3-phosphohydroxypyruvate (3-PHP) to 3-phosphoserine (PSer) in an L-glutamate (Glu)-linked reversible transamination reaction. This process proceeds through a bimolecular ping–pong mechanism and in plants takes place in plastids. It is a part of the phosphorylated pathway of serine biosynthesis, one of three routes recognized in plant organisms that yield serine. In this three-step biotransformation, 3-phosphoglycerate (3-PGA) delivered from plastidial glycolysis and Calvin cycle is oxidized by 3-PGA dehydrogenase. Then, 3-PHP is subjected to transamination with Glu to yield PSer and α-ketoglutarate (AKG). In the last step of the pathway, serine is produced by the action of phosphoserine phosphatase. Here we present the structural characterization of PSAT isoform 1 from *Arabidopsis thaliana* (*At*PSAT1), a dimeric S-shaped protein that truncated of its 71-residue-long chloroplast-targeting signal peptide. Three crystal structures of *At*PSAT1 captured at different stages of the reaction: (i) internal aldimine state with PLP covalently bound to the catalytic K265, (ii) holoenzyme in complex with pyridoxamine-5′-phosphate (PMP) after transfer of the amino group from glutamate and (iii) the geminal diamine intermediate state wherein the cofactor is covalently bound to both, K265 and PSer. These snapshots over the course of the reaction present detailed architecture of *At*PSAT1 and allow for the comparison of this plant enzyme with other PSATs. Conformational changes of the protein during the catalytic event concern (i) the neighborhood of K265 when the amino group is transferred to the cofactor to form PMP and (ii) movement of the gate-keeping loop (residues 391–401) upon binding of 3-PHP and PSer. The latter conformational change of the loop may likely be one of key elements that regulate catalytic activity of PSATs.

## Introduction

Serine is one of the endogenous proteinogenic amino acids which also acts as the precursor of glycine, tryptophan, and cysteine (Ireland and Hiltz, 1995). Moreover, it is an important intermediate in the biosynthesis of phospholipids, porphyrins, and nucleobases (Munoz-Bertomeu et al., 2013). Ser is also the major source of one-carbon units, essential for the methylation of nucleic acids and proteins (Ho and Saito, 2001).

Plant organisms have developed three independent routes for Ser biosynthesis (**Supplementary Figure S1**): two non-photorespiratory pathways, (i) phosphorylated and (ii) glycerate pathways, and also (iii) glycolate pathway that is linked with photorespiration (Ros et al., 2014). Both non-photorespiratory routes start with 3-PGA. In the phosphorylated pathway, which operates in plastids, 3-PGA is delivered from plastidial glycolysis and Calvin cycle. In contrast, the glycerate cycle is fed with 3-PGA from cytosolic glycolysis and enzymes of this pathway are localized in either cytosol or peroxisomes. Although both routes start with the same substrate, their organization is very different. The first reaction of phosphorylated pathway is NAD^+^-dependent oxidation of 3-PGA to 3-PHP by 3-PGA dehydrogenase. In the following step, PSer aminotransferase catalyzes transfer of amino group from Glu to 3-PHP to yield PSer and AKG. The last step involves cleavage of the PSer phosphate group by PSer phosphatase. In the glycerate pathway, the order of reactions is somewhat shuffled, with 3-PGA dephosphorylation followed by oxidation and finally aminotransfer. The glycolate route takes place in mitochondria, mostly within photosynthetic cells, whose activity is strongly connected with the day-night cycle (Cascales-Minana et al., 2013) and is regulated by the circadian clock (McClung et al., 2000). It starts with the biotransformation of two Gly molecules. The first molecule is utilized by GCS (Bauwe, 2017) which transforms THF to 5,10-CH_2_-THF. The reaction of 5,10-CH_2_-THF with the other Gly molecule, catalyzed by SHMT, yields Ser. In mitochondrion, direction of this thermodynamically unfavorable reaction toward Ser synthesis is attained through a high activity of the GCS, which delivers 5,10-CH_2_-THF for the SHMT reaction (Rebeille et al., 1994). However, plants have several isoforms of SHMT, localized in mitochondria, chloroplasts, nuclei, and cytoplasm (Ruszkowski et al., 2018). SHMTs outside the mitochondrial matrix preferentially catalyze a reversed Ser-to-Gly conversion to generate one-carbon units.

Plants have developed the alternate routes for Ser production probably because there was a necessity to bypass Ser biosynthesis through the glycolate pathway and make it independent of daylight to supply Ser equally to all tissues (Ros et al., 2014). Out of the two non-respiratory pathways, more Ser is produced through the phosphorylated pathway, which is important during the night and in non-photosynthetic tissues (Ros et al., 2014). Phosphorylated pathway activity regulates the glycolytic flux, affects the Krebs cycle and tryptophan biosynthesis rate (Munoz-Bertomeu et al., 2013). It is important for the plant development, especially at its early stages with respect to root formation (Benstein et al., 2013). Under environmental stress (high salinity, flooding or low temperature), the activity of phosphorylated pathway is alleviated which suggests that supplying with Ser is important for non-photosynthetic cells under harsh conditions (Ho and Saito, 2001). The phosphorylated pathway also affects other metabolic pathways like glycolysis, the tricarboxylic acid cycle, and amino acid biosynthesis (Toujani et al., 2013). Moreover, the phosphorylated pathway is induced upon infection of plants by pathogens (Benstein et al., 2013).

Phosphoserine aminotransferases belong to the class IV of ATs with the α-type fold (Liepman and Olsen, 2004). The α family of ATs predominantly catalyzes reactions at substrate Cα, that is the carbon atom of the substrate which is imine-bonded to PLP during the catalysis (Liepman and Olsen, 2004). PSATs are PLP-dependent enzymes that catalyze the second step of the phosphorylated pathway – reversible conversion of 3-PHP to PSer in a Glu-linked reaction (Basurko et al., 1999). In general, this class of enzymes is characterized by the presence of two domains with mixed α/β fold. Also, the conserved catalytic lysine, which directly follows a hydrophobic β-strand, is localized closer to the C-terminus than the Gly-rich region (Grishin et al., 1995). Another common feature of PSATs is an aspartate residue which hydrogen bonds the pyridoxal ring N1 atom and precedes the Schiff base lysine by 20–50 amino acids.

**FIGURE 1:**
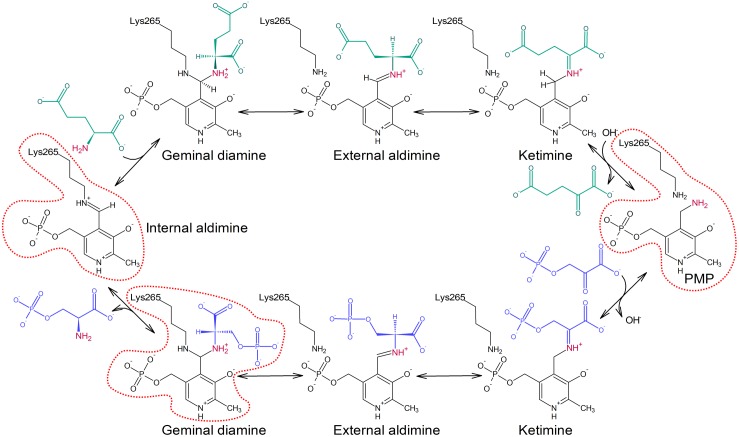
Schematic representation of the transamination reaction catalyzed by PSAT isoform 1 from *Arabidopsis thaliana* (*At*PSAT1). Reaction states presented in the crystallographic snapshots in this work are contoured with red dotted line.

The transamination reaction catalyzed by PSAT consists of two reversible half-reactions (**Figure 1**; Eliot and Kirsch, 2004). At the beginning of the catalytic event, the internal aldimine is protonated at Nζ of the catalytic lysine residue by the hydroxyl (O3) group of PLP which allows for the alignment of the imine and pyridine ring in a plane. In the first step, the amine nitrogen atom of Glu performs a nucleophilic attack on the iminium carbon of the internal aldimine and, through PLP-Glu geminal diamine intermediate, substitutes lysine Nζ with the creation of PLP-Glu external aldimine. Because PLP acts as an “electron sink,” it withdraws electrons from the intermediate and triggers the rearrangement of the double bond to form a ketimine. Afterwards, hydrolysis of the imine bond releases the newly created AKG. Now, the enzyme holding PMP is ready to accommodate 3-PHP, which then forms a covalent bond with PMP. The subsequent release of a water molecule produces another ketimine, and afterwards PLP-PSer external aldimine. Then, Nζ of the catalytic lysine performs a nucleophilic attack on the C4′ of PLP to form PLP-PSer geminal diamine intermediate. The reaction ends when the initial internal aldimine state is restored and PSer is released from the active site.

In this work, we describe a structure of the isoform 1 of chloroplastic PSAT from *A. thaliana* (*At*), which is further referred to in the manuscript as *At*PSAT1. The gene coding for *At*PSAT1 is localized in the lower arm of chromosome 4 and encodes the protein with an N-terminal plastidial transit peptide (Ho and Saito, 2001). *At*PSAT1 mRNA is expressed in all tissues with the highest expression level observed in the light-grown roots and shoots, with significantly lower rates of the dark-treated individuals (Ho et al., 1998). *At*PSAT1 is accumulated in the stele, especially in the cells close to the xylem in leaf, stem, and root sections. The full-length recombinant protein was reported previously to be inactive and insoluble, however, a variant truncated of approximately sixty N-terminal residues was active, with the *K*_M_-values for Glu and 3-PHP of 70 μM and 5 mM, respectively (Ho et al., 1998). The authors reported that cysteine at high concentration presented some inhibitory properties toward *At*PSAT1, but no inhibition was observed by 5–50 mM serine, threonine, valine, glycine, tryptophan, or *O*-acetyl-L-serine. Our crystal structures capture the enzyme with cleaved signal sequence in three different states, as: (i) internal aldimine with PLP covalently bound to the catalytic K265, (ii) the holoenzyme in complex with PMP after the transfer of the amino group from Glu, and (iii) the PLP-PSer geminal diamine intermediate wherein the cofactor is covalently bound to both K265 and to PSer. We discuss the structural adaptability of *At*PSAT1 over the course of the catalytic event and we also compare *At*PSAT1 structure with other ATs.

## Materials and Methods

### Cloning, Overexpression, and Purification of *At*PSAT1

*At*PSAT1 was cloned and purified using a modified protocol recently applied for the production of *Medicago truncatula* N-carbamoylputrescine amidohydrolase (Sekula et al., 2016) and thermospermine synthase (Sekula and Dauter, 2018). Isolation of total RNA from leaves of *A. thaliana* was performed with RNeasy Plant Mini Kit (Qiagen). SuperScript II reverse transcriptase (Life Technologies) and oligo dT (15 and 18) primers were used for the preparation of the complementary DNA (cDNA). The following primers, forward: TACTTCCAATCCAATGCCCGTGTCTTCAACTTCGCGGCG and reverse: TTATCCACTTCCAATGTTACTAAGCATGCTTAGCCTGGAAATCTTTC, and cDNA as a template were used in polymerase chain reaction to obtain the *At*PSAT1 open reading frame with the encoded protein starting from the codon number 72. The incorporation of *At*PSAT1 gene into pMCSG68 vector (Midwest Center for Structural Genomics) was performed according to the ligase-independent cloning protocol (Kim et al., 2011). The vector introduces N-terminal His_6_-tag followed by the Tobacco Etch Virus (TEV) protease cleavage site to the cloned protein. In the next step, the BL21 Gold *Escherichia coli* competent cells (Agilent Technologies) were transformed with the vector containing *At*PSAT1 gene. The cells were precultured at 30°C in LB medium with ampicillin (150 μg/ml) overnight. Next, 1.5% v/v of the culture was used as inoculum of the fresh LB medium with ampicillin. It was cultured at 37°C until OD_600_ reached the value of 1.0. In the next step, the culture was cooled to 10°C for 2 h and then the protein production was induced with 0.5 mM of isopropyl-β-D-thiogalactopyranoside. At this point K_2_HPO_4_ (40 mM) was added. The culture continued for 16 h at 18°C. Before pelleting the cells at 3,500 × g for 30 min, the culture was cooled to 4°C. The cell pellets were resuspended in 35 ml of the binding buffer (50 mM HEPES pH 7.4; 500 mM NaCl; 20 mM imidazole; 1 mM tris(2-carboxyethyl)phosphine, TCEP) and frozen at -80°C. Thawed cells were disrupted by sonication in an ice/water bath for 4 min (bursts of 4 s with 26 s intervals). Then, they were pelleted by centrifugation at 25,000 ×*g* for 30 min at 4°C.

The first step of *At*PSAT1 purification was performed using a column packed with 5 ml of HisTrap HP resin (GE Healthcare) connected to VacMan (Promega). The supernatant was applied to the column and washed five times with 40 ml of the binding buffer. The protein elution was performed with 20 ml of elution buffer (50 mM HEPES pH 7.4; 500 mM NaCl; 400 mM imidazole; 1 mM TCEP). His_6_-tagged TEV protease (final concentration of 0.1 mg/ml) was used to cleave the His_6_-tag from *At*PSAT1. This step was simultaneous to the overnight dialysis at 4°C against the dialysis buffer (50 mM HEPES pH 7.4; 500 mM NaCl; 1 mM TCEP). After dialysis, the sample was applied onto HisTrap HP resin to remove the cleaved His_6_-tag and TEV protease. The final step of the purification of *At*PSAT1 was size exclusion chromatography on HiLoad Superdex 200 16/60 column (GE Healthcare) connected to the AKTA FPLC system (Amersham Biosciences). The running buffer was 25 mM HEPES pH 7.4, 100 mM KCl, 50 mM NaCl, and 1 mM TCEP.

### Crystallization and Data Collection

*At*PSAT1 was concentrated with Amicon concentrators (Millipore) to the final concentration of approximately 15 mg/ml, determined by the absorbance measurement at 280 nm, with the extinction coefficient of 34380. Full saturation of the cofactor was obtained by addition of 1 mM PLP (final concentration) to the concentrated protein sample. *At*PSAT1 crystallized in the 77th condition of the Index Screen (Hampton Research), which contains 0.2 M lithium sulfate, 25% PEG 3350, and 0.1 M Tris at pH 8.5. Significantly better-diffracting crystals were obtained by streak seeding of the drops which were set up with the PEG concentration lowered to 17%. The protein was crystallized by sitting drop method. Such conditions were used for the crystallization of *At*PSAT1-PLP and *At*PSAT1-PMP complexes, but the *At*PSAT1-PMP complex was obtained by addition of 10 mM Glu to the protein sample before crystallization setup. The *At*PSAT1-PSer complex was cocrystallized with 50 mM PSer in 19% PEG 3350 and 0.1 M Tris at pH 8.5, without lithium sulfate. Crystals were transferred to the mixture of the well solution with 2-methyl-2,4-pentanediol at the volume ratio 2:1 to provide a sufficient cryoprotection before freezing in liquid nitrogen. In case of crystals of *At*PSAT1-PMP and *At*PSAT1-PSer, 10 mM Glu or 50 mM PSer were included in the cryoprotectant solution, respectively.

The diffraction data were collected at SER-CAT 22-ID and SBC 19-ID beamlines at the Advanced Photon Source (APS), Argonne National Laboratory, United States. The diffraction data were processed with *XDS* (Kabsch, 2010); for details see **Table 1**.

**Table 1:** Data-collection and refinement statistics.

Structure	*At*PSAT1-PLP	*At*PSAT1-PMP	*At*PSAT1-PSer
**Data collection**			
Beamline	19ID	19ID	22ID
Wavelength (Å)	0.979	0.979	1.0
Temperature (K)	100	100	100
Space group	*P*2_1_2_1_2_1_	*P*2_1_2_1_2_1_	*C*2
Unit cell parameters			
*a*, b, *c* (Å)	84.2, 105.9, 186.9	84.6, 106.5, 187.8	189.7, 53.3, 137.9
β (°)			91.9
Number of subunits in the asymmetric unit	4	4	4
Oscillation range (°)	0.5	0.5	0.5
Resolution (Å)	76.78–1.57 (1.66–1.57)	77.14–1.75 (1.85–1.75)	44.4–1.70 (1.80–1.70)
Reflections collected/unique	1703523/230909	1253772/170626	568810/149284
Completeness (%)	99.2 (98.1)	99.4 (96.6)	98.1 (95.6)
Multiplicity	7.4 (7.3)	7.3 (7.5)	3.8 (3.6)
*R*_merge_ (%)	8.9 (96.5)	10.2 (76.7)	8.0 (55.4)
< *I*/σ(*I)*>	13.33 (1.85)	14.3 (2.14)	10.1 (1.83)
*CC*_1/2_	99.9 (68.4)	99.8 (84.1)	99.7 (81.9)
**Refinement**			
*R*_free_ reflections	1155	1024	1190
No. of atoms (non-H)			
Protein	11492	11459	11310
Ligands	157	122	106
Solvent	1875	2031	1512
*R*_work_/*R*_free_ (%)	15.7/17.5	15.3/18.7	18.1/20.9
Mean ADP^a^ (Å^2^)			
Protein	21.4	22.2	25.8
Ligands	27.1	28.5	21.6
Solvent	32.0	33.8	33.5
RMSD from ideal geometry			
Bond lengths (Å)	0.01	0.01	0.01
Bond angles (^o^)	1.5	1.6	1.3
Ramachandran statistics (%)			
Favored	98	97	97
Allowed	2	3	3
Outliers	0	0	0
PDB code	6czx	6czy	6czz

*^a^ADP, atomic displacement parameter. Values in parentheses refer to the highest resolution shell*.

### Structure Determination and Refinement

The structure of *At*PSAT1 was solved by molecular replacement in *Phaser* (McCoy et al., 2007); the structure of *Pseudomonas aeruginosa* PSAT (*Pa*PSAT, PDB ID: 4xk1) was used as a search model. The initial model was rebuilt in *ARP/wARP* (Langer et al., 2008). Then, the structure was subjected to manual and automatic refinement with *Coot* (Emsley et al., 2010), *Refmac* (Murshudov et al., 2011), and *Phenix* (Adams et al., 2010). At the later stages of the structure refinement, *TLS* parameters (Winn et al., 2003) were applied. The refined structure of *At*PSAT1 was used as the model for determination of the structures of *At*PSAT1 with ligands. The stereochemical restraints for ligands were generated in *Sketcher* from CCP4 (Winn et al., 2011) and *Elbow* (Moriarty et al., 2009). The quality of refined structures was controlled by *R*_work_, *R*_free_ factors (Brunger, 1992) and geometric parameters. The evaluation of the final structures was performed in *PROCHECK* (Laskowski et al., 1993) and *MolProbity* (Chen et al., 2010). The final refinement statistics are given in **Table 1**.

### Other Software Used

Molecular illustrations were created with UCSF *Chimera* (Pettersen et al., 2004). Ramachandran plot was calculated in *Rampage* (Lovell et al., 2003). Secondary structure was recognized with *ProMotif* (Hutchinson and Thornton, 1996) within *PDBsum* server (de Beer et al., 2014). Sequence alignment was performed in *CLUSTAL W* (Thompson et al., 1994), and edited in *BioEdit* (Hall, 1999). Sequence conservation was calculated in *ConSurf* (Ashkenazy et al., 2016) based on the sequence alignment performed in *Muscle* (Edgar, 2004) under the *MEGA7* suite (Kumar et al., 2016) with sequences classified to the eukaryotic PSAT family (IPR022278) by *InterPro* (Finn et al., 2017). Surface electrostatic potential was calculated in *PDB2PQR* and *APBS* (Baker et al., 2001; Dolinsky et al., 2004).

## Results and Discussion

### *At*PSAT1 With the Fold of α-Type Aminotransferases

The subunit of *At*PSAT1 has the length of 430 residues, including the predicted N-terminal transit peptide. According to the previous reports, transit peptide with the length of 60- (Ho et al., 1998) or 76-residues (Liepman and Olsen, 2004) was predicted. The previous study (Ho et al., 1998) also confirmed that the full-length protein was insoluble and inactive, however, the construct truncated of about 60-residues regained its catalytic activity. An additional analysis in the *ChloroP 1.1.* server (Emanuelsson et al., 1999) indicated an 86-residues-long signal sequence, however, based on the sequence homology with different PSATs (**Supplementary Figure S2**) and the analysis of available structural data of PSATs from other kingdoms of life, the designed construct was truncated before residue R72. Thus, the expressed protein (residues 72–430) has the length of 359 residues, plus the N-terminal SNA tag (the remaining sequence after the TEV protease cleavage). Truncation of the 71-residue peptide seems to be adequate since the electron density maps for the N-terminal part of *At*PSAT1 structure is clearly defined (except for the SNA tag). On the other hand, a longer transit peptide is very doubtful – the polypeptide chain in the structure of *At*PSAT1 starts with a 12-residue long bent coil with a short fragment (residues 74–75) in β-strand conformation and reaches the PLP binding site around G79. Thus, a more pronounced truncation of the N-termini would most likely affect the active site of the enzyme.

**FIGURE 2:**
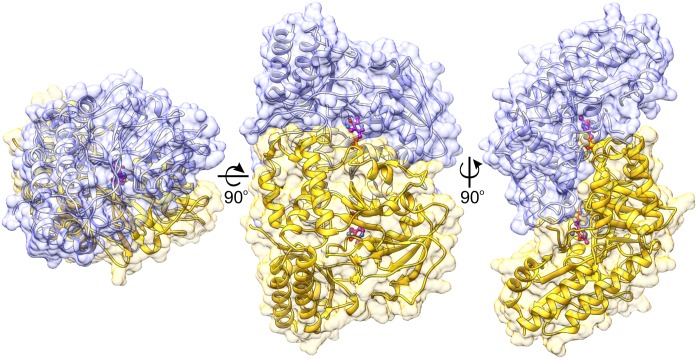
Biological assembly of *At*PSAT1 dimer. Chains A and B are colored in yellow and light blue; the bound cofactor (PMP) is shown in balls-and-sticks representation (magenta). Coloring is used consequently throughout the manuscript unless stated otherwise.

**FIGURE 3:**
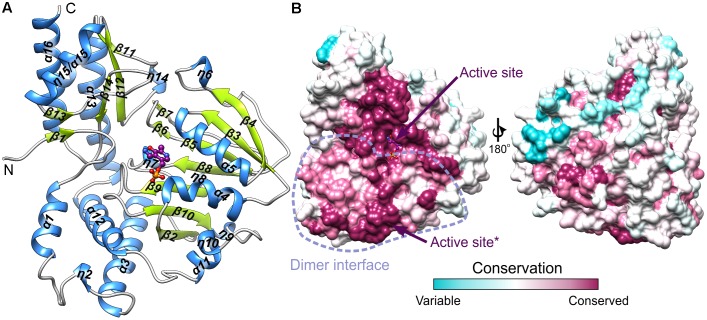
The subunit of *At*PSAT1. **(A)** Arrangement of the secondary structure elements of *At*PSAT1, helices (blue) and sheets (lime green); cofactor (PMP) is shown in balls-and-sticks representation (magenta). **(B)** Sequence conservation of the eukaryotic PSATs mapped the surface of *At*PSAT1 monomer with marked location of the active site and schematic boundaries of dimer interface; asterisk indicates active site of the other subunit of the dimer.

The protein mass calculated from the retention volume from size exclusion chromatography (∼80 kDa) and the crystal structure analyzed with PISA server (Krissinel, 2015) confirm the assembly of *At*PSAT1 to be homodimeric (**Figure 2**). Therefore, the asymmetric unit with four subunits represents two functional *At*PSAT1 dimers. The subunit of *At*PSAT1 consists of two domains with mixed α/β topology (**Figure 3A**). This is a typical fold of class IV of the α-type ATs with a much larger N-terminal catalytic domain and a smaller C-terminal domain. Hydrophobic core of the N-terminal domain is constituted by a seven-stranded β-sheet with one anti-parallel strand (↑β2-↓β10-↑β9-↑β8-↑β5-↑β3-↑β4). The sheet is covered from the inner side of the *At*PSAT1 dimer by helices α4, α5, η9, α10, α11, and by long loops from the outer side. An integral part of this domain is also a helical bundle (α1, η2, α3, α12) which builds a substantial part of the dimer interface. The small C-terminal domain comprises residues 326–430 which form a three-stranded anti-parallel β-sheet (↑β11-↓β12-↑β14) and a bundle of three helices (α13, η15/α15, α16). The symmetrical S-shaped dimer of *At*PSAT1 (**Figure 2**) is formed through an extensive interface area (**Figure 3B**) which is over 2300 Å^2^, or about 14% of the subunit surface. The dimer interface is created by residues from the N-terminal coil, α1, η2, the loop between α11 and α12, the loop after η9, η10, the loop between β9 and β10, and the helices α4 and α5. The small domain does not directly take part in formation of the *At*PSAT1 dimer, however, through a small β-sheet (↑β1-↑β13) it stabilizes the N-terminal coil. The dimer interface is rather flat with no significant cavities. Conservation of the residues at the interface is generally high among eukaryotic PSATs (**Figure 3B**). However, the highest conservation is, of course, observed around the PLP binding site, thus around the catalytic venue.

The Dali server (Holm and Rosenstrom, 2010) search through the Protein Data Bank (PDB) (Berman et al., 2000) shows a few protein structures with a significant sequence identity (**Supplementary Figure S2**), which are structurally very similar to *At*PSAT1: *Pa*PSAT (PDB ID: 4xk1, r.m.s.d. = 1.0 Å, *Z* = 53.8), *Yersinia pestis* PSAT (*Yp*PSAT, PDB ID: 3qbo, r.m.s.d. = 1.3 Å, *Z* = 51.9), *Homo sapiens* PSAT (*Hs*PSAT, PDB ID: 3e77, r.m.s.d. = 1.2 Å, *Z* = 51.2), *E. coli* PSAT (*Ec*PSAT, PDB ID: 1bjo, r.m.s.d. = 1.3 Å, *Z* = 52.0) (Hester et al., 1999), *Bacillus alcalophilus* PSAT (*Ba*PSAT, PDB ID: 4azj, r.m.s.d. = 1.1 Å, *Z* = 54.1) (Battula et al., 2013), *Salmonella enterica* (*Se*PSAT, PDB ID: 3qm2, r.m.s.d. = 1.4 Å, *Z* = 48.0). It is not surprising that the overall active site architecture is similar among PSATs of different species since they catalyze the same reaction. In all PSATs PLP is π-stacked with Trp (W171 in *At*PSAT1) that has been shown by mutagenesis to be necessary for the full PSAT activity (Mishra et al., 2012). However, a detailed look into other PSATs shows that the enzymes are not identical. For example, in the structure of *Hs*PSAT (PDB ID: 3e77), the N-terminal coil at first look seems to be placed significantly further from the active site, leaving about 6–7 Å free space in the neighborhood of the catalytic Lys. Longer coil in *At*PSAT1 has in this region P80 in *cis* conformation; the structure of *Hs*PSAT lacks it. In fact, the expressed *Hs*PSAT started with L17, thus at the end of the N-terminal coil. Residues 8–16 visible in the structure came from the expression tag. Sequence alignment (**Supplementary Figure S2**) and structural comparison of different PSATs allow to predict similar conformation of this coil to *At*PSAT1. Another distinguishing feature of *Hs*PSAT, is C80 corresponding to T146 of *At*PSAT1 which interacts with the phosphate group of PLP. In other PSATs there is Ser (in *Ba*PSAT and *Pa*PSAT) or Arg (*Ec*PSAT, *Se*PSAT, and *Yp*PSAT). Long sidechain of this Arg residue in *Ec*PSAT, *Se*PSAT, and *Yp*PSAT increases the positive charge around the substrate binding site and may directly take part in stabilization of the bound ligands, but it is unlikely to H-bond the phosphate of PLP. These three proteins also present significantly different conformation of the loop between α3 and β2, although this feature concerns region rather far away from the catalytic site. However, the D100A mutation of *Hs*PSAT (corresponding to D164 of *At*PSAT1), which is very close to this loop, causes significant solubility decrease and activity loss (Hart et al., 2007). Noteworthy, this mutation in *Hs*PSAT is one of the causes of the rare disorder named PSAT deficiency (Hart et al., 2007).

**FIGURE 4:**
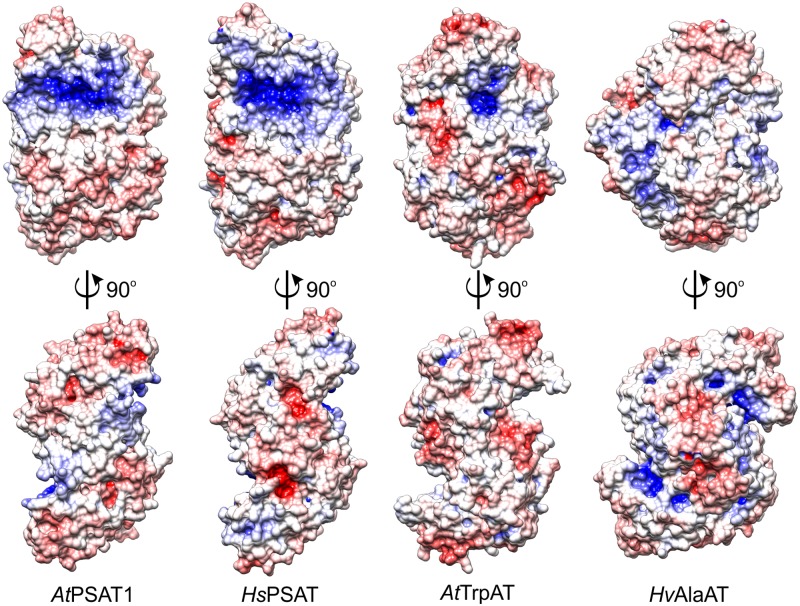
Charge distribution on the surface of selected aminotransferases (ATs): *A. thaliana* phosphoserine aminotransferase (*At*PSAT1, this work), *Homo sapiens* PSAT (*Hs*PSAT, PDB ID: 3e77), *A. thaliana* tryptophan aminotransferase (*At*TrpAT, PDB ID: 3bwn), *Hordeum vulgare* alanine aminotransferase (*Hv*AlaAT, PDB ID: 3tcm).

Among the plant ATs with structures deposited in the PDB there are two, although more divergent from *At*PSAT1: *A. thaliana* tryptophan aminotransferase (*At*TrpAT, PDB ID: 3bwn, r.m.s.d. = 3.4 Å, 14% sequence identity) (Tao et al., 2008) and *Hordeum vulgare* alanine aminotransferase (*Hv*AlaAT, PDB ID: 3tcm, r.m.s.d. = 3.3 Å, 13% identity) (Duff et al., 2012). The structural comparison of *At*PSAT1 with other structures of plant ATs shows that these proteins share the S-like shape of the dimer (**Figure 4**), although some, like *Hv*AlaAT, are bulkier, as if the “font” was changed. This is caused by the bigger C-terminal domains and additional helices in the PLP-binding domain, which in *Hv*AlaAT form a β-sandwich fold, not observed in PSATs. PSATs have fewer helical fragments in their catalytic domains than the standard of eight to nine helices in other ATs (John, 1995). ATs are most of the times dimers, however, there are examples of ATs which are active as tetramers, like the unusual Archean aspartate AT (Tanaka et al., 1992), or monomers, as for instance AlaAT from *Trypanosoma cruzi* (de Morais et al., 2016). The distinctive feature of PSATs, shared between species, is a rather open and vast, positively charged region, localized not only around the PLP-binding site, but also around the entire catalytic site (**Figure 4**). It is easily explainable by the necessity of PSATs to attract phosphorylated, thus negatively charged substrate, 3-PHP. In other plant ATs (*At*TrpAT and *Hv*AlaAT), which do not utilize phosphorylated substrates, this positively charged patch covers a much smaller area, only directly around the PLP-binding sites in a deep tunnel (*At*TrpAT) or is even completely covered by residues from the mostly helical extended N-terminus (*Hv*AlaAT, **Figure 4**).

### PLP Bound in the Active Site Forms Internal Aldimine With K265

**FIGURE 5:**
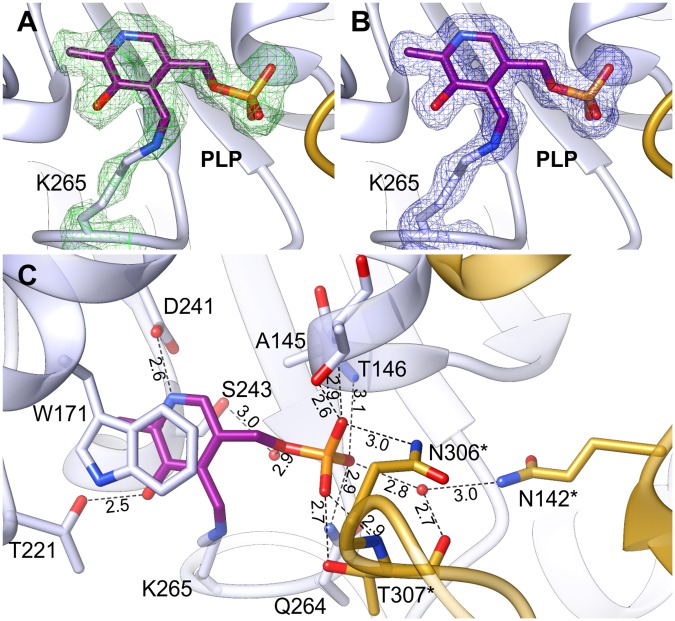
Internal aldimine in the *At*PSAT1-pyridoxal 5′-phosphate (PLP) structure. **(A)** OMIT *F*_o_*–F*_c_ electron density map for K265 and PLP contoured at 3σ; **(B)** 2*F*_o_*–F*_c_ map electron density map for K265 and PLP contoured at 1σ. **(C)** A detailed PLP binding mode; asterisks indicate residues from the other subunit.

The positive electrostatic potential around the catalytic venue is achieved by the contribution of K174, H396, R397, R403, and R110^∗^, H111^∗^ (asterisks indicate residues from the other subunit within the dimer), and secures the binding of both, PLP and the negatively charged substrates. The two active sites in the dimer are distant from each other by about 30 Å and are placed in the cavities on the opposite sides of the dimer (**Figure 2**). The PLP prosthetic group creates a Schiff base (internal aldimine) between C4′ and Nζ of K265 (**Figures 5A,B**), the residue placed in the coil between β9 and β10. Most of the residues interacting with PLP are placed on the C-terminal sides of the β-strands of the large domain core. PLP is bound inside the active pocket with its pyridoxal ring π-stacked with W171 on the *re*-face of the cofactor (**Figure 5C**). The protonated N1 of the pyridoxal ring is H-bonded to the carboxylate of D241. Another hydrogen bond is created between the hydroxyl group of PLP and T221. Phosphate of PLP creates a much more pronounced hydrogen bonding network with the protein. It includes direct H-bonds with backbone amides of A145 and T146, which, due to the placement at the N-terminal part of the α4 helix, possess an increased positive charge. Hydrogen bonds of PLP phosphate group also involve interactions with the side chain amide of Q264 and the hydroxyl group of T146. The phosphate also forms contacts with residues of the *At*PSAT1 dimer mate, particularly hydrogen bonds with: the side chain amide of N306^∗^, the main chain amide of T307^∗^, and the side chain hydroxyl group of T307^∗^. Moreover, the phosphate of PLP creates water-mediated hydrogen bonds with the hydroxyl group of S243, the backbone amide of G262, the carbonyl oxygen of T307^∗^ and N𝜀 of Q142^∗^. Altogether, before the catalytic event, the PLP prosthetic group in *At*PSAT1 structure presents a very conserved conformation in the internal aldimine state similar to that observed in other ATs, where C4′ atom of PLP is exposed to the entrance of the pocket.

### Complex With PMP Shows the Enzyme Primed for a Covalent Binding of 3-PHP

**FIGURE 6:**
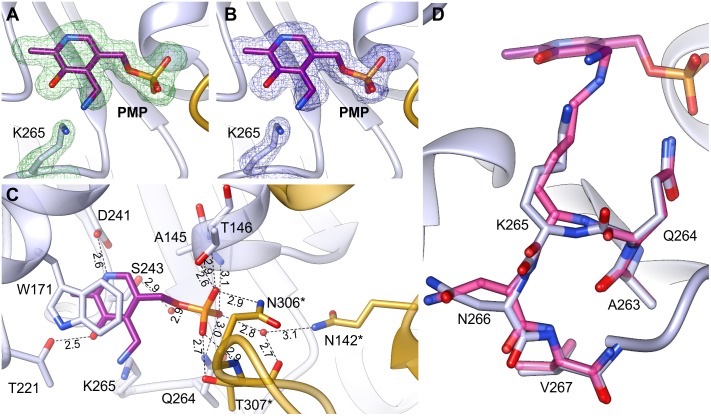
*At*PSAT1-PMP complex. **(A)** OMIT *F*_o_*–F*_c_ electron density map for K265 and PMP contoured at 3σ; **(B)** 2*F*_o_*–F*_c_ map electron density map for K265 and PMP contoured at 1σ. **(C)** A detailed PMP binding mode; asterisks indicate residues from the other subunit. **(D)** Comparison of the conformation of *At*PSAT1-PMP (light blue) with *At*PSAT1-PLP internal aldimine (pink).

The *At*PSAT1-PMP complex was obtained by crystallization of *At*PSAT1-PLP with Glu. Neither Glu nor AKG (after the transfer of the amino group to PLP) were present in the crystal structure. However, electron density maps (**Figures 6A,B**) revealed the cofactor with the amino group already transferred from Glu and no longer covalently bound to K265, clearly showing that the first half of transamination reaction had taken place. The overall *At*PSAT1-PMP structure is very similar to *At*PSAT1-PLP (r.m.s.d. = 0.4 Å). Position of PMP is almost identical as PLP, thus its interactions with the protein are preserved (**Figure 6C**), excluding of course the broken covalent bond with K265. Position of the amine transferred to C4′ is almost in plane with the PMP pyridoxal ring, where it creates an intramolecular H-bond with the hydroxyl group of PMP. Although there is no movement of the prosthetic group observed during the transfer of the amino group from Glu and transition from the internal aldimine state to form PMP, the protein modifies conformation in the neighborhood of K265. The change concerns the main chain of Q264 and residues K265 and N266 (**Figure 6D**). The side chain of K265 is more bent with the Nζ amine moved by about 2 Å from its position in *At*PSAT1-PLP structure. The Cα position of K265 and N265 is shifted away from the PMP by about 0.7 Å. This conformational change influences the position of the carbonyl oxygen of Q264 (which is rotated toward PMP by over 30°) and the conformation of N266 side chain as well. Such adaptation of the protein allows to achieve the position of the cofactor amino group exposed slightly toward the entrance of the active site. This way, PMP is ready for a nucleophilic attack on the Cα of 3-PHP bound in the active site in the next stage of the transamination reaction.

### *At*PSAT1-PSer Structure Brings Insights Into the Unexpected Geminal Diamine Intermediate State

**FIGURE 7:**
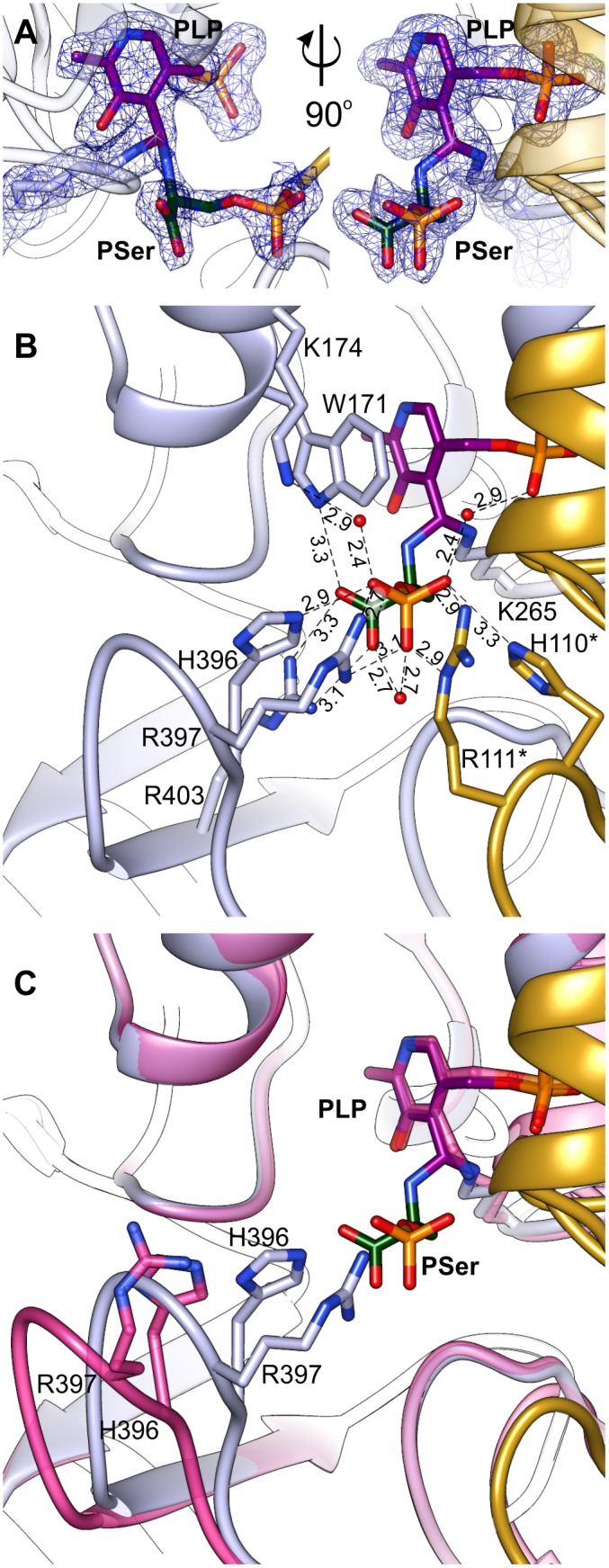
Geminal diamine in *At*PSAT1-PSer structure. **(A)** 2*F*_o_*–F*_c_ map electron density map for K265, PLP, and PSer contoured at 1σ. **(B)** A detailed PSer binding mode; asterisks indicate residues from the other subunit. **(C)** Comparison of the 391–401 loop conformation in *At*PSAT1-PSer structure (light blue) and *At*PSAT1-PLP structure (pink).

Initial unsuccessful attempts to determine the structure of *At*PSAT1-PSer complex resulted in a very “blurry” difference electron density maps around the catalytic site. Finally, crystallization of *At*PSAT1 with 50 mM PSer and without sulfate ions in the crystallization conditions succeeded and the structure has shown somewhat unexpected results. The electron density maps (**Figure 7A** and **Supplementary Figure S3**) revealed not only the position of PSer in the active site itself, but they also indicated the presence of two covalent bonds: between the nitrogen atom of PSer and C4′ of PLP, and between C4′ of PLP and Nζ atom of K265. Thus, the structure does not show neither the last step of the transamination reaction, in the internal aldimine state (with PSer non-covalently bound just before its release from the active site) nor the PLP-PSer external aldimine. The reaction snapshot in the *At*PSAT1-PSer structure actually shows one of the reaction intermediates – the PLP-PSer geminal diamine (**Figure 1**).

In this complex, the prosthetic group, similarly to the other structures (*At*PSAT1-PLP and *At*PSAT1-PMP) preserves interactions with the protein, with a maintained position of the phosphate group. The pyridoxal ring presents a subtle in-plane rotation (by about 8°). As a result of creation of two covalent bonds, the C4′ atom of PLP presents sp^3^ hybridization. In the PLP-PSer geminal diamine state, K265 and its neighborhood present almost identical conformation as in the internal aldimine state (the *At*PSAT1-PLP structure). The bound PSer creates several hydrogen bonds in the active site, which include: a salt bridge between PSer carboxylate and guanidine group of R403 placed in the β14 strand, an H-bond between one of the oxygen atoms from the carboxyl group of PSer and N𝜀 of the indole ring of W171 from the α5 helix. Oxygen atoms from the phosphate group of PSer create several hydrogen bonds, either directly with protein side chains or through water molecules (**Figure 7B**). These include direct hydrogen bonds with H396, R397 (loop between β13 and β14), and with R111^∗^, H110^∗^ (loop between η2 and α3). The water-mediated hydrogen bonds include interaction with K174, H-bond with the carboxyl group of PSer, and interaction with oxygen from PLP phosphate group.

The superposition of *At*PSAT1-PLP and *At*PSAT1-PSer shows a pronounced movement of the loop 391–401 toward the catalytic site associated with PSer binding (**Figure 7C**). This clearly shows that during the binding of phosphorylated ligands (3-PHP or PSer) this gate-keeping loop transits from open to close conformation in order to stabilize the ligand during the amino group transfer, in a very similar way to the structure of *Ba*PSAT (PDB ID: 4azj), where authors captured PLP-PSer external aldimine state (Battula et al., 2013). Therefore, it is very likely that sulfate ions, which competed with PSer to interact with H396, R397, R111^∗^, and H110^∗^, prevented from stable PSer binding in the initial crystallization attempts of *At*PSAT1-PSer complex.

Previous report shows that the transamination reaction catalyzed by PSAT is able to proceed in both directions (Basurko et al., 1999) and the donor of the amino group can be Glu or PSer. Of course, kinetics and thermodynamics of the reversed reaction may be completely different than physiological transamination with Glu and 3-PHP in the direction of PSer synthesis. We observe PLP-PSer geminal diamine in our structure, which suggests a relative stability of this intermediate state. It is, therefore, very likely that the direction of Ser production is controlled by the activity of PSer phosphatase, catalyzing the final and irreversible step of Ser synthesis (Wang et al., 2002).

### Binding the Substrates Requires a Conformational Change of *At*PSAT1

It has been proposed that ATs reach their full catalytic activity only in the closed conformation (John, 1995). Taking this into account, a requirement for the catalytic event to occur is that the loop 391–401 of *At*PSAT1 has to adopt an optimal conformation, with H396 and R397 poised closer to the PLP binding site to direct the substrates in the appropriate position (**Figure 7C**). This suggests that open-to-close transition of the 391–401 loop most likely guides the substrates toward the catalytic site. It is worth noting that the electron density maps for this region in the *At*PSAT1-PSer structure clearly show the position for residues within this loop. In case of the other two structures, the electron density maps are blurrier and with no meaningful signal for the side chain of R397, which indicates a flexibility of this region. Since binding of PSer apparently stabilizes conformation of the gate-keeping loop, it is very likely that a similar stabilization secures 3-PHP as well. Such a bracing would therefore promote the catalytic event by locking the substrate in an optimal position.

## Conclusion

The presented in this work crystal structures of *At*PSAT1 bring detailed insights into the conversion of 3-PHP to PSer. Therefore, they expand the knowledge about the phosphorylated pathway of serine metabolism in plants. *At*PSAT1 is a dimeric PLP-dependent AT that, similarly to other α-type ATs, binds the prosthetic group in the cavity within the large domain. The three crystal structures of *At*PSAT1 present snapshots along the catalytic event showing PLP internal aldimine state, complex with PMP after the first half-reaction and PSer-PLP geminal diamine intermediate state. The structures give detailed information about the reaction catalyzed by *At*PSAT1, revealing structural changes in the neighborhood of the catalytic K265 during the transition from the internal aldimine to the PMP complex. Moreover, the structure of *At*PSAT1-PSer shows the closed conformation of the gate-keeping loop between β13 and β14 (residues 391–401), required for the stabilization of *At*PSAT1-PSer geminal diamine and most likely for the catalysis as well. The transition from open to close conformation of the gate-keeping loop probably improves loading of 3-PHP and discarding PSer after the catalysis. *At*PSAT1-PSer crystal structure additionally shows that the reverse reaction follows only to the PLP-PSer geminal diamine intermediate state, at least in the crystalline state.

## Accession Numbers

Coordinates and structure factors of the related structures were deposited in the Protein Data Bank: 6czx (*At*PSAT1-PLP), 6czy (*At*PSAT1-PMP), 6czz (*At*PSAT1-PSer).

## Author Contributions

BS and MR planned and performed the experiments, analyzed the results, and wrote the manuscript. ZD analyzed the results and supervised the work.

## Conflict of Interest Statement

The authors declare that the research was conducted in the absence of any commercial or financial relationships that could be construed as a potential conflict of interest.

## Abbreviations

3-PGA: 3-phosphoglycerate
3-PHP: 3-phosphohydroxypyruvate
5,10-CH_2_-THF: *N*^5^,*N*^10^-methylene-tetrahydrofolate
AKG: α-ketoglutarate
AT: aminotransferase
GCS: glycine cleavage system
PLP: pyridoxal 5′-phosphate
PMP: pyridoxamine-5′-phosphate
PSAT: phosphoserine aminotransferase
PSer: 3-phosphoserine
SHMT: serine hydroxymethyltransferase
THF: tetrahydrofolate

## References

[B1] Adams P. D. Afonine P. V. Bunkoczi G. Chen V. B. Davis I. W. Echols N. (2010). PHENIX: a comprehensive Python-based system for macromolecular structure solution. *Acta Crystallogr. D* 66 213–221. 10.1107/S0907444909052925 20124702 PMC2815670

[B2] Ashkenazy H. Abadi S. Martz E. Chay O. Mayrose I. Pupko T. (2016). ConSurf 2016: an improved methodology to estimate and visualize evolutionary conservation in macromolecules. *Nucleic Acids Res.* 44 W344–W350. 10.1093/nar/gkw408 27166375 PMC4987940

[B3] Baker N. A. Sept D. Joseph S. Holst M. J. McCammon J. A. (2001). Electrostatics of nanosystems: application to microtubules and the ribosome. *Proc. Natl. Acad. Sci. U.S.A.* 98 10037–10041. 10.1073/pnas.181342398 11517324 PMC56910

[B4] Basurko M. J. Marche M. Darriet M. Cassaigne A. (1999). Phosphoserine aminotransferase, the second step-catalyzing enzyme for serine biosynthesis. *IUBMB Life* 48 525–529. 10.1080/713803557 10637769

[B5] Battula P. Dubnovitsky A. P. Papageorgiou A. C. (2013). Structural basis of L-phosphoserine binding to Bacillus alcalophilus phosphoserine aminotransferase. *Acta Crystallogr. D* 69 804–811. 10.1107/s0907444913002096 23633589

[B6] Bauwe H. (2017). Measurement of enzyme activities. *Methods Mol. Biol.* 1653 31–50. 10.1007/978-1-4939-7225-8_3 28822124

[B7] Benstein R. M. Ludewig K. Wulfert S. Wittek S. Gigolashvili T. Frerigmann H. (2013). *Arabidopsis* phosphoglycerate dehydrogenase1 of the phosphoserine pathway is essential for development and required for ammonium assimilation and tryptophan biosynthesis. *Plant Cell* 25 5011–5029. 10.1105/tpc.113.118992 24368794 PMC3904002

[B8] Berman H. M. Westbrook J. Feng Z. Gilliland G. Bhat T. N. Weissig H. (2000). The protein data bank. *Nucleic Acids Res.* 28 235–242. 10.1093/nar/28.1.235 10592235 PMC102472

[B9] Brunger A. T. (1992). Free R value: a novel statistical quantity for assessing the accuracy of crystal structures. *Nature* 355 472–475. 10.1038/355472a0 18481394

[B10] Cascales-Minana B. Munoz-Bertomeu J. Flores-Tornero M. Anoman A. D. Pertusa J. Alaiz M. (2013). The phosphorylated pathway of serine biosynthesis is essential both for male gametophyte and embryo development and for root growth in *Arabidopsis*. *Plant Cell* 25 2084–2101. 10.1105/tpc.113.112359 23771893 PMC3723614

[B11] Chen V. B. Arendall W. B. Headd J. J. Keedy D. A. Immormino R. M. Kapral G. J. (2010). MolProbity: all-atom structure validation for macromolecular crystallography. *Acta Crystallogr. D* 66 12–21. 10.1107/S0907444909042073 20057044 PMC2803126

[B12] de Beer T. A. Berka K. Thornton J. M. Laskowski R. A. (2014). PDBsum additions. *Nucleic Acids Res.* 42 D292–D296. 10.1093/nar/gkt940 24153109 PMC3965036

[B13] de Morais S. B. de Arruda Campos Brasil de Souza T. Weiler A. V. Murakami M. T. (2016). Biophysical characterization of alanine aminotransferase from *Trypanosoma cruzi*. *Protein Pept. Lett.* 23 1118–1122. 10.2174/0929866523666161025120511 27781954

[B14] Dolinsky T. J. Nielsen J. E. McCammon J. A. Baker N. A. (2004). PDB2PQR: an automated pipeline for the setup of Poisson-Boltzmann electrostatics calculations. *Nucleic Acids Res.* 32 W665–W667. 10.1093/nar/gkh381 15215472 PMC441519

[B15] Duff S. M. Rydel T. J. McClerren A. L. Zhang W. Li J. Y. Sturman E. J. (2012). The enzymology of alanine aminotransferase (AlaAT) isoforms from *Hordeum vulgare* and other organisms, and the HvAlaAT crystal structure. *Arch. Biochem. Biophys.* 528 90–101. 10.1016/j.abb.2012.06.006 22750542

[B16] Edgar R. C. (2004). MUSCLE: multiple sequence alignment with high accuracy and high throughput. *Nucleic Acids Res.* 32 1792–1797. 10.1093/nar/gkh340 15034147 PMC390337

[B17] Eliot A. C. Kirsch J. F. (2004). Pyridoxal phosphate enzymes: mechanistic, structural, and evolutionary considerations. *Annu. Rev. Biochem.* 73 383–415. 10.1146/annurev.biochem.73.011303.074021 15189147

[B18] Emanuelsson O. Nielsen H. von Heijne G. (1999). ChloroP, a neural network-based method for predicting chloroplast transit peptides and their cleavage sites. *Protein Sci.* 8 978–984. 10.1110/ps.8.5.978 10338008 PMC2144330

[B19] Emsley P. Lohkamp B. Scott W. G. Cowtan K. (2010). Features and development of Coot. *Acta Crystallogr. D* 66 486–501. 10.1107/S0907444910007493 20383002 PMC2852313

[B20] Finn R. D. Attwood T. K. Babbitt P. C. Bateman A. Bork P. Bridge A. J. (2017). InterPro in 2017-beyond protein family and domain annotations. *Nucleic Acids Res.* 45 D190–D199. 10.1093/nar/gkw1107 27899635 PMC5210578

[B21] Grishin N. V. Phillips M. A. Goldsmith E. J. (1995). Modeling of the spatial structure of eukaryotic ornithine decarboxylases. *Protein Sci.* 4 1291–1304. 10.1002/pro.5560040705 7670372 PMC2143167

[B22] Hall T. A. (1999). BioEdit: a user-friendly biological sequence alignment editor and analysis program for Windows 95/98/NT. *Nucleic Acids Symp. Ser.* 41 95–98.

[B23] Hart C. E. Race V. Achouri Y. Wiame E. Sharrard M. Olpin S. E. (2007). Phosphoserine aminotransferase deficiency: a novel disorder of the serine biosynthesis pathway. *Am. J. Hum. Genet.* 80 931–937. 10.1086/517888 17436247 PMC1852735

[B24] Hester G. Stark W. Moser M. Kallen J. Markovic-Housley Z. Jansonius J. N. (1999). Crystal structure of phosphoserine aminotransferase from *Escherichia coli* at 2.3 A resolution: comparison of the unligated enzyme and a complex with alpha-methyl-l-glutamate. *J. Mol. Biol.* 286 829–850. 10.1006/jmbi.1998.2506 10024454

[B25] Ho C. L. Noji M. Saito M. Yamazaki M. Saito K. (1998). Molecular characterization of plastidic phosphoserine aminotransferase in serine biosynthesis from *Arabidopsis*. *Plant J.* 16 443–452. 10.1046/j.1365-313x.1998.00313.x 9881164

[B26] Ho C. L. Saito K. (2001). Molecular biology of the plastidic phosphorylated serine biosynthetic pathway in *Arabidopsis thaliana*. *Amino Acids* 20 243–259. 10.1007/s007260170042 11354602

[B27] Holm L. Rosenstrom P. (2010). Dali server: conservation mapping in 3D. *Nucleic Acids Res.* 38 W545–W549. 10.1093/nar/gkq366 20457744 PMC2896194

[B28] Hutchinson E. G. Thornton J. M. (1996). PROMOTIF–a program to identify and analyze structural motifs in proteins. *Protein Sci.* 5 212–220. 10.1002/pro.5560050204 8745398 PMC2143354

[B29] Ireland R. J. Hiltz D. A. (1995). “Glycine and serine synthesis in non-photosynthetic tissues,” in *Amino Acids and their Derivatives in Higher Plants*, ed. Wallsgrove R. M. (Cambridge: Cambridge University Press), 111–118. 10.1017/CBO9780511721809.009

[B30] John R. A. (1995). Pyridoxal phosphate-dependent enzymes. *Biochim. Biophys. Acta* 1248 81–96. 10.1016/0167-4838(95)00025-P 7748903

[B31] Kabsch W. (2010). Xds. *Acta Crystallogr. D* 66 125–132. 10.1107/S0907444909047337 20124692 PMC2815665

[B32] Kim Y. Babnigg G. Jedrzejczak R. Eschenfeldt W. H. Li H. Maltseva N. (2011). High-throughput protein purification and quality assessment for crystallization. *Methods* 55 12–28. 10.1016/j.ymeth.2011.07.010 21907284 PMC3690762

[B33] Krissinel E. (2015). Stock-based detection of protein oligomeric states in jsPISA. *Nucleic Acids Res.* 43 W314–W319. 10.1093/nar/gkv314 25908787 PMC4489313

[B34] Kumar S. Stecher G. Tamura K. (2016). MEGA7: molecular evolutionary genetics analysis version 7.0 for bigger datasets. *Mol. Biol. Evol.* 33 1870–1874. 10.1093/molbev/msw054 27004904 PMC8210823

[B35] Langer G. Cohen S. X. Lamzin V. S. Perrakis A. (2008). Automated macromolecular model building for X-ray crystallography using ARP/wARP version 7. *Nat. Protoc.* 3 1171–1179. 10.1038/nprot.2008.91 18600222 PMC2582149

[B36] Laskowski R. A. Macarthur M. W. Moss D. S. Thornton J. M. (1993). Procheck - a program to check the stereochemical quality of protein structures. *J. Appl. Crystallogr.* 26 283–291. 10.1107/S0021889892009944

[B37] Liepman A. H. Olsen L. J. (2004). Genomic analysis of aminotransferases in *Arabidopsis thaliana*. *Crit. Rev. Plant Sci.* 23 73–89. 10.1080/07352680490273419

[B38] Lovell S. C. Davis I. W. Arendall W. B. III de Bakker P. I. Word J. M. Prisant M. G. (2003). Structure validation by Calpha geometry: phi,psi and Cbeta deviation. *Proteins* 50 437–450. 10.1002/prot.10286 12557186

[B39] McClung C. R. Hsu M. Painter J. E. Gagne J. M. Karlsberg S. D. Salome P. A. (2000). Integrated temporal regulation of the photorespiratory pathway. Circadian regulation of two Arabidopsis genes encoding serine hydroxymethyltransferase. *Plant Physiol.* 123 381–392. 10.1104/pp.123.1.381 10806255 PMC59012

[B40] McCoy A. J. Grosse-Kunstleve R. W. Adams P. D. Winn M. D. Storoni L. C. Read R. J. (2007). Phaser crystallographic software. *J. Appl. Crystallogr.* 40 658–674. 10.1107/s0021889807021206 19461840 PMC2483472

[B41] Mishra V. Kumar A. Ali V. Nozaki T. Zhang K. Y. J. Bhakuni V. (2012). Role of conserved active site tryptophan-101 in functional activity and stability of phosphoserine aminotransferase from an enteric human parasite. *Amino Acids* 43 483–491. 10.1007/s00726-011-1105-x 22038178

[B42] Moriarty N. W. Grosse-Kunstleve R. W. Adams P. D. (2009). Electronic ligand builder and optimization workbench (eLBOW): a tool for ligand coordinate and restraint generation. *Acta Crystallogr. D* 65 1074–1080. 10.1107/S0907444909029436 19770504 PMC2748967

[B43] Munoz-Bertomeu J. Anoman A. Flores-Tornero M. Toujani W. Rosa-Tellez S. Fernie A. R. (2013). The essential role of the phosphorylated pathway of serine biosynthesis in *Arabidopsis*. *Plant Signal. Behav.* 8:e27104. 10.4161/psb.27104 24299976 PMC4091574

[B44] Murshudov G. N. Skubak P. Lebedev A. A. Pannu N. S. Steiner R. A. Nicholls R. A. (2011). REFMAC5 for the refinement of macromolecular crystal structures. *Acta Crystallogr. D* 67 355–367. 10.1107/S0907444911001314 21460454 PMC3069751

[B45] Pettersen E. F. Goddard T. D. Huang C. C. Couch G. S. Greenblatt D. M. Meng E. C. (2004). UCSF Chimera–a visualization system for exploratory research and analysis. *J. Comput. Chem.* 25 1605–1612. 10.1002/jcc.20084 15264254

[B46] Rebeille F. Neuburger M. Douce R. (1994). Interaction between glycine decarboxylase, serine hydroxymethyltransferase and tetrahydrofolate polyglutamates in pea leaf mitochondria. *Biochem. J.* 302 223–228. 10.1042/bj3020223 7520695 PMC1137213

[B47] Ros R. Munoz-Bertomeu J. Krueger S. (2014). Serine in plants: biosynthesis, metabolism, and functions. *Trends Plant Sci.* 19 564–569. 10.1016/j.tplants.2014.06.003 24999240

[B48] Ruszkowski M. Sekula B. Ruszkowska A. Dauter Z. (2018). Chloroplastic serine hydroxymethyltransferase from *Medicago truncatula*: a structural characterization. *Front. Plant Sci.* 9:584. 10.3389/fpls.2018.00584 29868052 PMC5958214

[B49] Sekula B. Dauter Z. (2018). Crystal structure of thermospermine synthase from *Medicago truncatula* and substrate discriminatory features of plant aminopropyltransferases. *Biochem. J.* 475 787–802. 10.1042/bcj20170900 29367265 PMC7983153

[B50] Sekula B. Ruszkowski M. Malinska M. Dauter Z. (2016). Structural investigations of N-carbamoylputrescine amidohydrolase from *Medicago truncatula*: insights into the ultimate step of putrescine biosynthesis in plants. *Front. Plant Sci.* 7:350. 10.3389/fpls.2016.00350 27066023 PMC4812014

[B51] Tanaka T. Yamamoto S. Taniguchi M. Hayashi H. Kuramitsu S. Kagamiyama H. (1992). Further studies on aspartate aminotransferase of thermophilic methanogens by analysis of general properties, bound cofactors, and subunit structures. *J. Biochem.* 112 811–815. 10.1093/oxfordjournals.jbchem.a123981 1295891

[B52] Tao Y. Ferrer J. L. Ljung K. Pojer F. Hong F. Long J. A. (2008). Rapid synthesis of auxin via a new tryptophan-dependent pathway is required for shade avoidance in plants. *Cell* 133 164–176. 10.1016/j.cell.2008.01.049 18394996 PMC2442466

[B53] Thompson J. D. Higgins D. G. Gibson T. J. (1994). CLUSTAL W: improving the sensitivity of progressive multiple sequence alignment through sequence weighting, position-specific gap penalties and weight matrix choice. *Nucleic Acids Res.* 22 4673–4680. 10.1093/nar/22.22.4673 7984417 PMC308517

[B54] Toujani W. Muñoz-Bertomeu J. Flores-Tornero M. Rosa-Téllez S. Anoman A. D. Alseekh S. (2013). Functional characterization of the plastidial 3-phosphoglycerate dehydrogenase family in Arabidopsis. *Plant Physiol.* 163 1164–1178. 10.1104/pp.113.226720 24058165 PMC3813641

[B55] Wang W. Cho H. S. Kim R. Jancarik J. Yokota H. Nguyen H. H. (2002). Structural characterization of the reaction pathway in phosphoserine phosphatase: crystallographic “snapshots” of intermediate states. *J. Mol. Biol.* 319 421–431. 10.1016/s0022-2836(02)00324-8 12051918

[B56] Winn M. D. Ballard C. C. Cowtan K. D. Dodson E. J. Emsley P. Evans P. R. (2011). Overview of the CCP4 suite and current developments. *Acta Crystallogr. D* 67 235–242. 10.1107/S0907444910045749 21460441 PMC3069738

[B57] Winn M. D. Murshudov G. N. Papiz M. Z. (2003). Macromolecular TLS refinement in REFMAC at moderate resolutions. *Methods Enzymol.* 374 300–321. 10.1016/S0076-6879(03)74014-2 14696379

