# Supplementary material for: Structural Analysis of Phosphoserine Aminotransferase (Isoform 1) From Arabidopsis thaliana– the Enzyme Involved in the Phosphorylated Pathway of Serine Biosynthesis
Source: Front Plant Sci. 2018 Jul 6;9:876. doi: 10.3389/fpls.2018.00876 (PMC6043687; doi:10.3389/fpls.2018.00876)
Supplement: Supplementary file 1 [file Image_1.PDF]

Supplementary Material

Structural analysis of phosphoserine aminotransferase (isoform 1) from *Arabidopsis thaliana*– the enzyme involved in the phosphorylated pathway of serine biosynthesis

Bartosz Sekula*, Milosz Ruszkowski and Zbigniew Dauter

Synchrotron Radiation Research Section of Macromolecular Crystallography Laboratory,
National Cancer Institute, Argonne, IL, USA

Corresponding author:
Bartosz Sekula
bartosz.sekula@nih.gov

Supplementary Figures:

Figure S1. Serine biosynthesis pathways in plants.

Figure S2. Sequence alignment of selected PSATs.

Figure S3. Geminal diamine in *At*PSAT1-PSer structure: OMIT electron density maps.

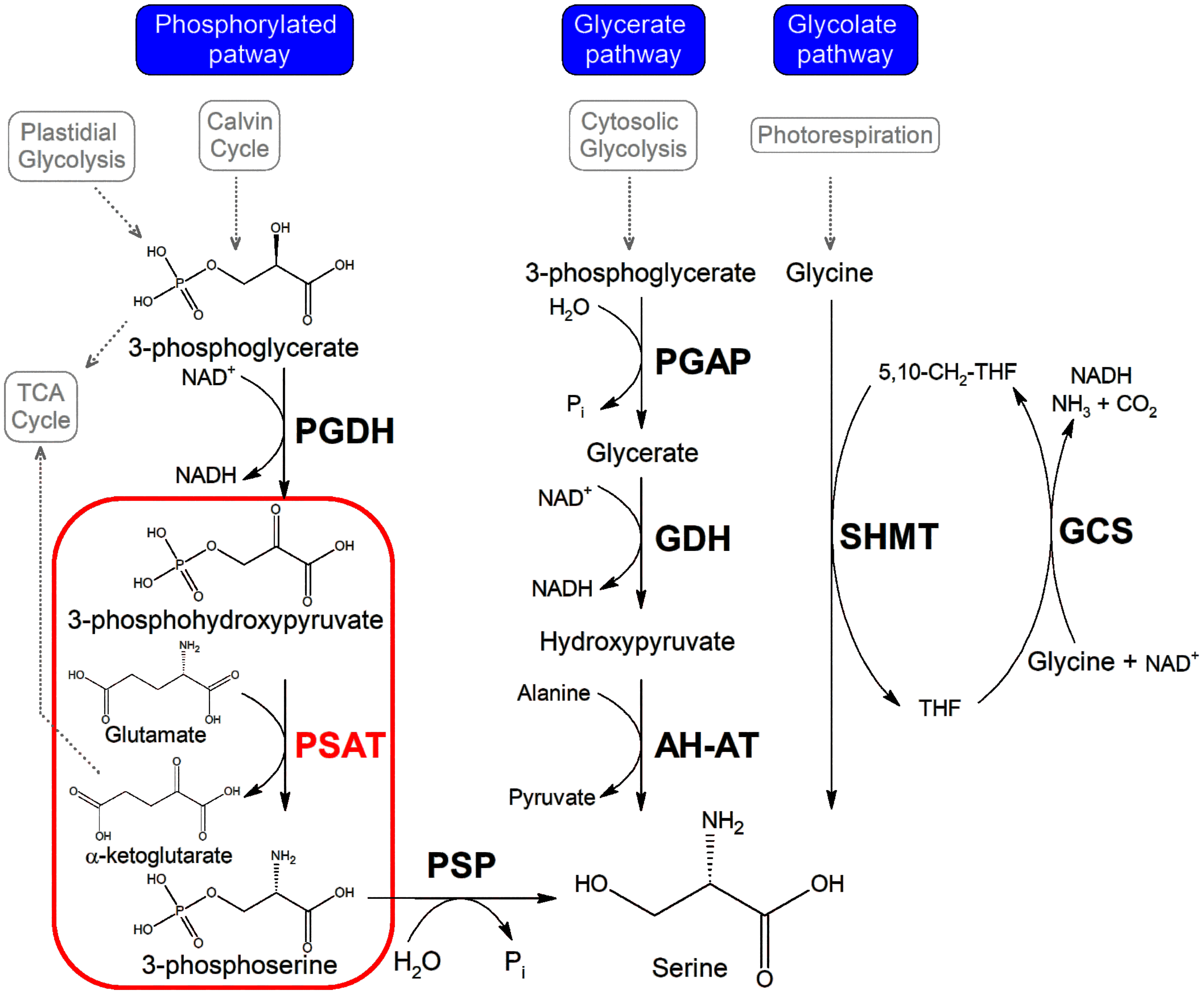

Supplementary figure S1

Serine biosynthesis pathways in plants. The reaction catalyzed by PSAT is marked in red. Abbreviations for the enzymes are as follows: PGDH, 3-phosphoglycerate dehydrogenase; PSAT, 3-phosphoserine aminotransferase; PSP, 3-phosphoserine phosphatase; PGAP, 3-phosphoglycerate phosphatase; GDH, glycerate dehydrogenase; AH-AT, alanine-hydroxypyruvate aminotransferase, GCS, glycine cleavage system; SHMT, serine hydroxymethyltransferase. Dashed lines indicate the source of particular substrates or an alternative direction of reaction.

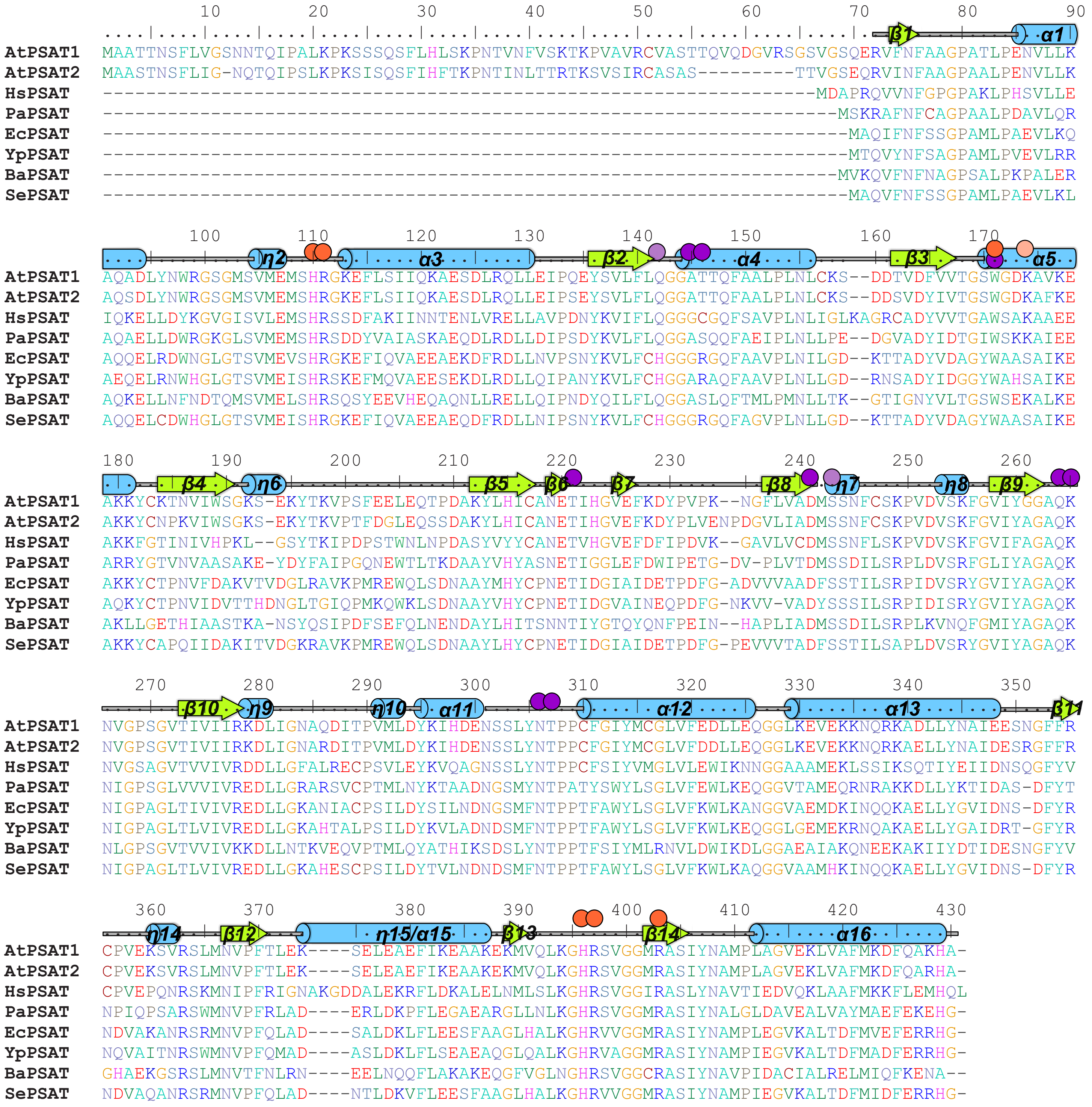

Supplementary figure S2

Sequence alignment of selected PSATs. UniProt accession numbers are given in square brackets, and the values in percent indicate sequence identity to *At*PSAT1 [Q96255] (transit peptides were excluded from the identity calculation). *At*PSAT2 [Q9SHP0, 91.4% sequence identity], *Hs*PSAT [Q9Y617, 49.9%], *Pseudomonas aeruginosa* PSAT [Q9HZ66, 51.4%], *Escherichia coli* PSAT [P23721, 48.5%], *Yersinia pestis* PSAT [Q8ZGB4, 43.0%], *Bacillus alcalophilus* PSAT [Q9RME2, 45.0%], *Salmonella enterica* [P55900, 47.9%]. Numbering above the sequences and annotation of the secondary structure elements (α helices and 3_{10} helices, η, are shown as blue cylinders and β strands are shown as lime green arrows) corresponds to *At*PSAT1. Residues are color-coded by type. Violet and orange circles indicate residues interacting with the cofactor and PSer, respectively. Residues participating in water-mediated interactions with ligands are shown in lighter shades.

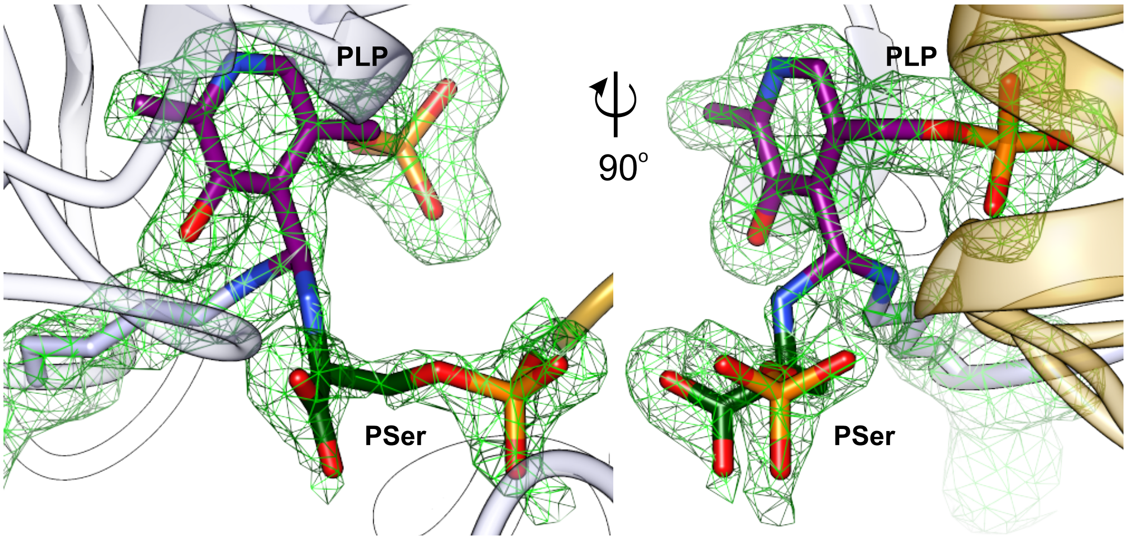

Supplementary figure S3

Geminal diamine in *At*PSAT1-PSer structure. OMIT F_o-F_c electron density maps (green mesh) are contoured at 3 σ level.
